# Orthodontic Treatment-Induced Periodontal, Microbiological, and Local Inflammatory Changes: A Systematic Review and Meta-Analysis

**DOI:** 10.3390/biomedicines14061308

**Published:** 2026-06-09

**Authors:** Dragos-Mihai Gavrilescu, Diana-Maria Mateescu, Andrei Marginean, Cristina Tudoran, Adrian-Cosmin Ilie, Marius Badalica-Petrescu, Dan Alexandru Surducan, Eduard Florescu, Raul Tirinescu, Ioana Cotet, Florin Eugen Constantinescu, Alina Tischer, Camelia-Oana Muresan

**Affiliations:** 1 Department of Orthodontics, Dental District, Strada Zăgazului Nr. 3, One Floreasca Vista, Sector 1, 014261 Bucharest, Romania; dr.gavrilescu@outlook.com; 2 Doctoral School, Department of General Medicine, “Victor Babes” University of Medicine and Pharmacy, Eftimie Murgu Square 2, 300041 Timisoara, Romania; diana.mateescu@umft.ro (D.-M.M.); ioana.cotet@umft.ro (I.C.); 3 Department of Surgery, Dr. Victor Popescu Emergency Military Hospital, 9 Gheorghe Lazăr Street, 300080 Timisoara, Romania; 4 Centre of Molecular Research in Nephrology and Vascular Disease, University of Medicine and Pharmacy “Victor Babes” Timisoara, E. Murgu Square, Nr. 2, 300041 Timisoara, Romania; tudoran.cristina@umft.ro; 5 Department VII, Internal Medicine II, Discipline of Cardiology, University of Medicine and Pharmacy “Victor Babes” Timisoara, E. Murgu Square, Nr. 2, 300041 Timisoara, Romania; 6 County Emergency Hospital “Pius Brinzeu”, L. Rebreanu, Nr. 156, 300723 Timisoara, Romania; 7 Department of Public Health and Sanitary Management, “Victor Babes” University of Medicine and Pharmacy, Eftimie Murgu Square 2, 300041 Timisoara, Romania; surducan.dan@umft.ro; 8 Centre for Translational Research and Systems Medicine, Faculty of Medicine, “Victor Babes” University of Medicine and Pharmacy, Eftimie Murgu Sq. No. 2, 300041 Timisoara, Romania; 9 Cardiology Department, “Victor Babes” University of Medicine and Pharmacy, Eftimie Murgu Square 2, 300041 Timisoara, Romania; 10 Pulmonology Department, “Victor Babes” University of Medicine and Pharmacy, 300041 Timisoara, Romania; 11 Oncology Department, M Hospital, Strada Witting 10–12, 010903 Bucharest, Romania; tirinescuraul@gmail.com; 12 Department of Prosthodontics, Faculty of Dental Medicine, Carol Davila University of Medicine and Pharmacy, 8 Eroii Sanitari Blvd, 050474 Bucharest, Romania; dr.florin.constantinescu@gmail.com; 13 Ear-Nose-Throat Department, “Victor Babes” University of Medicine and Pharmacy Timisoara, 300041 Timisoara, Romania; tischer.alina@umft.ro; 14 Legal Medicine, Timisoara Institute of Legal Medicine, 300041 Timisoara, Romania; muresan.camelia@umft.ro; 15 Ethics and Human Identification Research Center, “Victor Babes” University of Medicine and Pharmacy, Eftimie Murgu Square 2, 300041 Timisoara, Romania; 16 Discipline of Forensic Medicine, Bioethics, Deontology, and Medical Law, Department of Neuroscience, “Victor Babes” University of Medicine and Pharmacy, Eftimie Murgu Square 2, 300041 Timisoara, Romania

**Keywords:** orthodontic appliances, fixed appliances, clear aligners, periodontal health, oral microbiota, gingival crevicular fluid, interleukin-1β, interleukin-6, systematic review, meta-analysis

## Abstract

**Background/Objectives**: Orthodontic treatment induces controlled mechanical forces that alter the periodontal environment, including changes in oral microbiota composition and activation of local inflammatory pathways. Despite the widespread and growing use of orthodontic appliances across all age groups, the magnitude, timing, and multi-domain biological impact of these changes have not been comprehensively quantified in a single systematic synthesis. This systematic review and meta-analysis aimed to synthesize the available evidence on periodontal clinical parameters, oral microbiota composition, and local inflammatory biomarkers associated with orthodontic treatment using fixed appliances and clear aligners, and to provide a structured, GRADE-rated evidence base for clinical practice. **Methods**: A systematic review and meta-analysis was conducted in accordance with PRISMA 2020 guidelines. PubMed/MEDLINE, Scopus, and Web of Science were searched from inception to March 2026. Prospective cohort studies, longitudinal clinical studies, and randomized controlled trials evaluating periodontal parameters, oral microbiota, and inflammatory biomarkers during orthodontic treatment were included. Quantitative synthesis was performed using mean differences or standardized mean differences with 95% confidence intervals, primarily assessing within-group (pre–post) changes. **Results**: Eighteen studies (*n* = 812 patients; follow-up 3–12 months) met inclusion criteria. Fixed orthodontic appliances were consistently associated with transient increases in plaque index (MD 0.45, 95% CI 0.32–0.58; I^2^ = 62%), gingival index (MD 0.38, 95% CI 0.25–0.51; I^2^ = 55%), and bleeding on probing (MD 15.2%, 95% CI 10.1–20.3%; I^2^ = 48%), particularly during early treatment phases. Microbiological analyses demonstrated within-group shifts toward increased prevalence of periodontopathogenic species (*Streptococcus mutans* OR 2.45, 95% CI 1.89–3.18; *Porphyromonas* spp. OR 2.14, 95% CI 1.67–2.75) in patients treated with fixed appliances. Local inflammatory responses were characterized by elevated IL-1β (MD 1.2, 95% CI 0.8–1.6) and IL-6 (MD 0.9, 95% CI 0.6–1.2) in gingival crevicular fluid. Certainty of evidence was rated moderate for plaque and gingival indices and low for microbiological and inflammatory outcomes (GRADE). **Conclusions**: Orthodontic treatment—particularly with fixed appliances—is associated with transient, reversible deterioration of periodontal indices, shifts toward a more dysbiotic oral microbiome, and elevation of local inflammatory mediators in gingival crevicular fluid during active treatment phases. These changes are manageable through structured preventive protocols and regular periodontal monitoring. Future prospective studies with concurrent control groups and standardized multi-domain outcome measures are needed to better define the magnitude and reversibility of these biological responses. PROSPERO: CRD420261336117.

## 1. Introduction

Orthodontic treatment is among the most frequently performed interventions in modern dentistry. Its primary aims are to correct malocclusion, improve masticatory function, and enhance facial esthetics and oral health-related quality of life [1,2]. The use of fixed orthodontic appliances and clear aligners has expanded substantially across all age groups, including adults [2]. Despite well-established clinical benefits, orthodontic therapy may significantly alter the oral environment by creating retentive niches for bacterial biofilm accumulation. This leads to changes in periodontal status, oral microbiota composition, and local inflammatory responses within periodontal tissues [3,4,5].

Dental plaque tends to accumulate around brackets, wires, and aligner attachments, resulting in temporary increases in plaque index, gingival inflammation, and bleeding on probing—even in patients with adequate oral hygiene [3,4]. Orthodontic appliances can impair effective plaque removal and disrupt the ecological balance of the oral cavity [6,7]. Furthermore, orthodontic tooth movement triggers complex biological processes, including periodontal ligament remodeling, alveolar bone resorption and apposition, and activation of inflammatory pathways involving cytokines, prostaglandins, and matrix metalloproteinases [8,9].

The clinical relevance of these orthodontic-induced biological changes extends beyond transient gingival discomfort. Epidemiological data indicate that a substantial proportion of orthodontic patients—particularly adults—develop clinically significant gingival inflammation within the first months of treatment, with some progressing to early attachment loss if preventive monitoring is inadequate [3,5]. The expanding use of orthodontic therapy in adult populations, who frequently present with pre-existing subclinical periodontal vulnerability, heightens the importance of accurately characterizing the magnitude and reversibility of these biological changes. Moreover, the oral microbiota alterations induced by fixed appliances—including increased colonization by cariogenic and periodontopathogenic species—may persist beyond the initial inflammatory phase and contribute to long-term ecological imbalance within the oral cavity [6,7]. Quantifying these multi-domain biological responses is therefore not merely of academic interest but directly relevant to clinical decision-making regarding preventive protocols, appliance selection, and periodontal monitoring frequency throughout orthodontic therapy.

Despite the extensive clinical use of orthodontic appliances, the biological responses induced in periodontal tissues have not been comprehensively synthesized within a multi-domain, quantitative framework. Previous systematic reviews have typically addressed individual outcome domains in isolation—either periodontal clinical parameters or microbiological changes—but no prior meta-analysis has simultaneously quantified the magnitude, timing, and inter-relationships of periodontal deterioration, microbiota shifts, and local inflammatory mediator elevation across comparable studies. This gap is clinically relevant: a concurrent characterization of these three inter-related biological processes is essential to determine whether orthodontic treatment poses a meaningful risk to periodontal health and whether preventive protocols should be intensified, particularly in adults with pre-existing periodontal vulnerability or cardiometabolic comorbidities [10,11,12].

Several systematic reviews have examined individual aspects of orthodontic-induced biological changes but with important limitations. Reviews focused on periodontal outcomes have generally not incorporated concurrent microbiological or inflammatory biomarker data, while microbiome-oriented studies have rarely contextualized their findings within the framework of clinical periodontal indices or gingival crevicular fluid cytokine profiles [3,4,5]. Furthermore, the majority of existing syntheses lack structured quality assessment using validated frameworks such as GRADE, limiting the interpretability and clinical applicability of their conclusions. No prior meta-analysis has simultaneously quantified periodontal clinical deterioration, oral microbiota shifts, and local inflammatory mediator elevation across a comparable set of prospective studies, nor has any previous review performed domain-stratified quantitative synthesis with explicit separation of within-group and between-group effect estimates. This methodological gap substantially constrains the ability of clinicians to assess the overall biological burden of orthodontic treatment and to identify patient subgroups—such as adults with treated periodontitis or metabolic comorbidities—who may require intensified preventive management during active therapy [10,11,12].

Moreover, although local inflammation is a well-established and necessary driver of orthodontic tooth movement at the cellular level, the aggregate quantitative evidence regarding the magnitude, temporal pattern, and reversibility of clinically measurable inflammatory changes across patient populations remains limited and fragmented. Existing studies use heterogeneous outcome measures and frequently lack structured quality assessment. Therefore, a methodologically rigorous systematic review and meta-analysis adhering to PRISMA 2020 standards, incorporating GRADE evidence rating, and performing domain-stratified quantitative synthesis is warranted to provide a reliable evidence base for clinical practice and future research.

Beyond local periodontal changes, a growing body of literature has established associations between periodontal inflammation and systemic inflammatory responses, including elevations in circulating C-reactive protein and pro-inflammatory cytokines [10,11,12]. Whether orthodontic treatment, by transiently modifying the periodontal environment and oral microbiota, may also influence systemic inflammatory markers remains poorly characterized. Given the clinical selection criterion that patients with untreated moderate-to-severe periodontitis are generally not eligible for orthodontic treatment, any systemic effects are expected to be limited; however, this question has not been systematically addressed.

The present systematic review and meta-analysis aimed to synthesize the available evidence on periodontal clinical parameters, oral microbiota composition, and local inflammatory biomarkers associated with orthodontic treatment using fixed appliances and clear aligners, and to provide a comprehensive multi-domain evidence base with structured quality assessment to inform clinical practice and future research directions.

## 2. Materials and Methods

### 2.1. Study Design and Reporting Standards

This systematic review and meta-analysis was designed and reported in accordance with the Preferred Reporting Items for Systematic Reviews and Meta-Analyses (PRISMA 2020) statement and its updated methodological guidance for evidence synthesis research [13]. The review protocol was developed a priori to ensure methodological transparency and reproducibility.

To minimize reporting bias and enhance transparency in evidence synthesis, the protocol was prospectively registered in the International Prospective Register of Systematic Reviews (PROSPERO) database: CRD420261336117. Prospective registration of systematic review protocols has been recommended to prevent duplication of research efforts and selective reporting of outcomes [14]. The registered protocol includes the predefined primary outcomes and eligibility criteria, which cover periodontal, microbiological, and local inflammatory parameters assessed during orthodontic treatment.

The PRISMA 2020 checklist is provided in the Supplementary Table S1.

All primary outcomes (periodontal, microbiological, and local inflammatory parameters) and subgroup analyses according to orthodontic appliance type were prespecified in the registered protocol. Where individual included studies additionally reported systemic inflammatory biomarkers (e.g., serum hs-CRP), these observations are noted descriptively in a separate exploratory subsection at the end of the Discussion, clearly distinguished from the pre-specified outcomes, and are not included in the primary synthesis or conclusions.

### 2.2. Eligibility Criteria

Eligibility criteria were established using the PICOS framework (Population, Intervention, Comparison, Outcomes, Study Design), which is widely recommended for systematic reviews in clinical research [15].

Population: Patients undergoing orthodontic treatment with fixed orthodontic appliances, multibracket systems, or removable aligner therapy.

Intervention: Orthodontic treatment involving mechanical tooth movement induced by orthodontic appliances.

Comparison: Baseline clinical measurements before orthodontic treatment, longitudinal follow-up measurements during orthodontic therapy, or comparisons between different orthodontic appliance types.

Outcomes: Studies were eligible if they reported at least one of the following pre-specified primary outcomes: periodontal clinical parameters (e.g., plaque index, gingival index, bleeding on probing, probing depth); microbiological alterations in oral or subgingival microbiota; local inflammatory biomarkers (e.g., cytokines in gingival crevicular fluid, including IL-1β and IL-6). Studies reporting systemic inflammatory markers (e.g., serum C-reactive protein) in addition to at least one primary outcome were eligible; systemic data are presented descriptively as exploratory observations only, separate from the primary synthesis.

Study Design: Eligible study designs included: prospective cohort studies, longitudinal clinical studies, randomized controlled clinical trials. The following were excluded: narrative reviews, systematic reviews and meta-analyses, case reports, conference abstracts, animal or in vitro studies, studies lacking original clinical data.

Given the anticipated clinical and methodological heterogeneity in study populations, designs, and outcome measures, outcomes were analyzed separately by domain (periodontal, microbiological, inflammatory), and quantitative synthesis was restricted to sufficiently homogeneous subsets of studies, complemented by qualitative synthesis where appropriate.

### 2.3. Information Sources and Search Strategy

A comprehensive electronic search was conducted in the following databases: PubMed/MEDLINE; Scopus; and Web of Science.

The search covered all records from database inception until the most recent update prior to the analysis.

The search strategy incorporated both controlled vocabulary terms and free-text keywords related to orthodontic treatment, periodontal health, oral microbiota, and inflammatory biomarkers. Boolean operators (“AND”, “OR”) were used to combine search terms and maximize retrieval sensitivity.

The primary search strategy combined orthodontic treatment terms with microbiota- and inflammation-related terms: (“orthodontic treatment” OR “orthodontic appliances” OR “clear aligners”) AND (“oral microbiota” OR “oral microbiome”) AND (“cytokines” OR “inflammatory biomarkers”). To increase sensitivity and avoid omission of clinically relevant orthodontic studies primarily focused on periodontal parameters, this strategy was supplemented with additional free-text terms related to periodontal outcomes (e.g., “plaque index”, “gingival index”, “bleeding on probing”, “probing depth”) and general inflammatory processes. Studies were eligible if they reported at least one of the predefined outcomes (periodontal, microbiological, or inflammatory), regardless of whether all three outcome domains were assessed.

The complete search strategies used for each database are provided in Supplementary Table S2. In addition to the electronic database search, the reference lists of all included studies and relevant systematic reviews identified during screening were manually screened to identify potentially eligible studies not captured by the database searches. No restrictions were applied regarding language of publication; however, all studies identified during the final selection were published in English, and no non-English records met inclusion criteria. The potential for language bias therefore cannot be fully excluded: studies reporting negative or null findings published in non-English journals may be underrepresented in the three searched databases, which could have introduced a directional bias toward studies with positive results. Gray literature sources (conference proceedings, theses, unpublished trial registries such as ClinicalTrials.gov or ISRCTN) were not formally searched. This decision was made on pragmatic grounds, as gray literature in this specific clinical domain (orthodontic biology, periodontal biomarkers) is rarely indexed or consistently accessible, and pilot hand-searches did not identify additional eligible records. However, this approach carries the inherent risk that small negative studies, which are less likely to be published in indexed journals, may be absent from the synthesis, potentially inflating effect size estimates. This limitation is explicitly acknowledged and discussed in Section 4.7. Given that fewer than ten studies contributed to most individual analyses, formal assessment of publication bias through funnel plots or Egger’s test was not methodologically appropriate and was therefore not performed, in accordance with current Cochrane guidance.

### 2.4. Study Screening and Selection Process

The screening process was conducted independently by two reviewers. Titles and abstracts were initially screened to identify potentially eligible studies. Full-text articles were subsequently assessed for inclusion by the same two reviewers. Any disagreements were resolved through discussion, and when necessary, by consultation with a third reviewer.

The study selection process followed the PRISMA 2020 flow diagram methodology [13].

### 2.5. Data Extraction

Data extraction was performed independently by two reviewers using a standardized template. Extracted data were cross-checked, and discrepancies were resolved through discussion and consensus. The following variables were extracted: first author and year of publication, study design, sample size, patient demographics, type of orthodontic appliance, duration of follow-up, periodontal clinical parameters, microbiological outcomes, inflammatory biomarkers.

When studies reported outcomes as medians with interquartile ranges or ranges, means and standard deviations were estimated using established methods as recommended by the Cochrane Handbook. When numerical data were not explicitly reported, values were extracted from figures where possible using digital extraction tools (WebPlotDigitizer, version 4.6). This approach was applied to a minority of studies and may have introduced additional imprecision in the extracted values compared to directly reported numerical data. The potential impact on pooled estimates is acknowledged as a limitation, and relevant outcomes are noted accordingly. In cases of missing or unclear data, no imputation beyond standard conversion methods was performed.

### 2.6. Risk-of-Bias Assessment

The methodological quality of included studies was evaluated using structured risk-of-bias assessment approaches recommended for systematic reviews. Randomized controlled trials were assessed using validated risk-of-bias frameworks, while observational studies were evaluated according to methodological criteria addressing selection bias, measurement bias, confounding, and completeness of follow-up [16].

Disagreements in methodological assessment were resolved through discussion and consensus. Risk-of-bias assessment was performed independently by two reviewers, with disagreements resolved through discussion or consultation with a third reviewer.

For randomized trials, domain-level judgments were made according to the RoB 2 tool across bias domains (randomization process, deviations from intended interventions, missing outcome data, measurement of outcomes, and selection of reported results). For observational studies, the Newcastle–Ottawa Scale domains (selection, comparability, and outcome assessment) were evaluated. Risk-of-bias assessments were considered qualitatively in the interpretation of pooled results.

### 2.7. Certainty of Evidence

The overall certainty of evidence across studies was evaluated using the GRADE (Grading of Recommendations Assessment, Development and Evaluation) framework [17]. This methodology assesses the certainty of evidence based on several domains, including risk of bias, inconsistency, indirectness, imprecision, and potential publication bias.

Evidence quality was categorized as high, moderate, low, or very low according to GRADE recommendations. The results of the GRADE assessment are summarized in Supplementary Table S3.

### 2.8. Statistical Analysis

Quantitative synthesis was conducted when studies reported sufficiently comparable outcomes. Continuous variables were pooled using mean differences (MD) or standardized mean differences (SMD) with corresponding 95% confidence intervals (CI).

Statistical heterogeneity was assessed using the I^2^ statistic, with values of 25%, 50%, and 75% representing low, moderate, and high heterogeneity, respectively. Random-effects models (DerSimonian–Laird method) were applied when heterogeneity was moderate to high (I^2^ > 50%), whereas fixed-effect models were used when heterogeneity was low.

Subgroup analyses were considered according to the following factors: type of orthodontic appliance (fixed appliances vs. clear aligners), type of outcome (periodontal, microbiological, inflammatory), duration of orthodontic treatment.

Meta-regression and extensive sensitivity analyses were not performed due to the limited number of studies available for several outcomes, as methodological guidance for evidence synthesis recommends caution when fewer than ten studies are available for reliable meta-regression analyses.

All statistical analyses were performed using Review Manager (RevMan), version 5.4 (Cochrane Collaboration, London, UK).

The choice between mean difference (MD) and standardized mean difference (SMD) was determined a priori based on the measurement scale and variability of each outcome across studies. Within-group (pre–post) and between-group comparisons were analyzed separately where appropriate. Odds ratios reported in microbiological analyses reflect pooled estimates of pathogen prevalence within each appliance category rather than direct comparisons between groups. These odds ratios should be interpreted descriptively and not as direct comparative effect estimates between appliance types. Due to heterogeneity in study design, outcome definitions, and follow-up duration, results were interpreted cautiously and supported by qualitative synthesis. For some outcomes, effect sizes differed across studies (MD vs. SMD) due to variations in measurement scales. To avoid inappropriate comparisons, results were presented separately by effect size type.

Assessment of publication bias was not formally performed due to the limited number of studies available for most outcomes (<10 studies per analysis), in accordance with methodological recommendations. Therefore, no funnel plots or formal statistical tests were conducted.

For periodontal outcomes, mean differences (MD) were used when studies reported outcomes on the same scale, whereas standardized mean differences (SMD) were applied when outcome scales differed across studies. This explains the use of different effect measures across appliance groups in pooled tables.

Within-group (pre–post) changes and between-group comparisons were analyzed separately and are presented descriptively within the same tables for clarity, rather than as direct pooled comparisons between appliance types.

Standard forest plots including individual study-level effect estimates are provided in Supplementary Figures S1–S3.

Due to the limited number of studies reporting systemic biomarkers, no separate meta-analysis for systemic outcomes was performed, and these findings were synthesized descriptively.

## 3. Results

### 3.1. Study Selection

The literature search identified a total of 1248 records across the selected electronic databases, including 312 records from PubMed/MEDLINE, 486 from Scopus, and 450 from Web of Science. After removal of duplicate records, 812 articles remained for title and abstract screening. Following the initial screening process, 142 full-text articles were assessed for eligibility.

**Figure 1:**
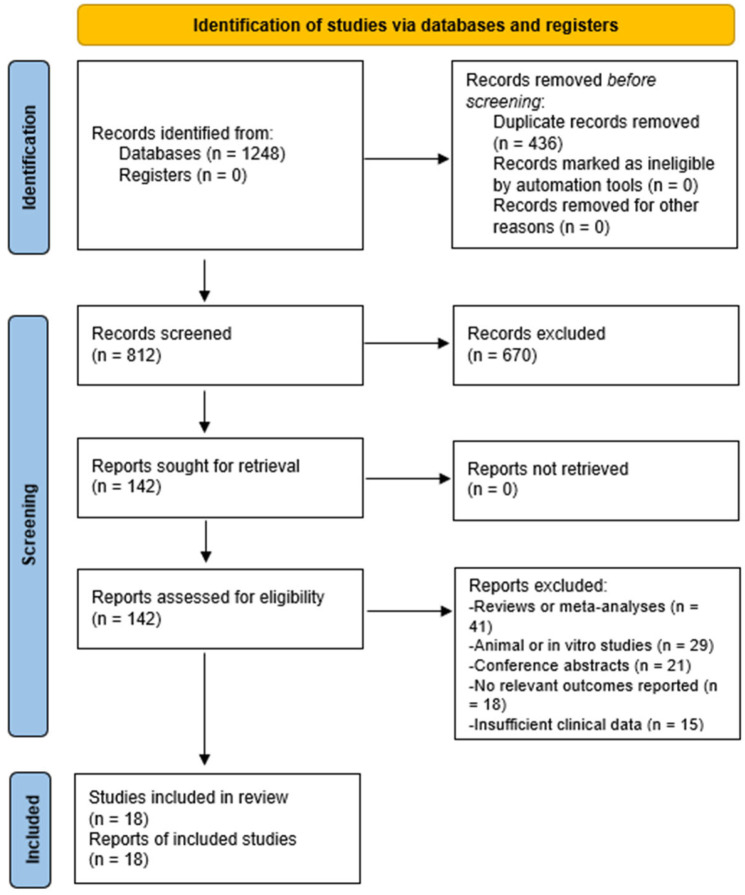
PRISMA 2020 flow diagram of the study selection process. The database search identified 1248 records from PubMed/MEDLINE, Scopus, and Web of Science. After duplicate removal, 812 records were screened by title and abstract. A total of 142 full-text articles were assessed for eligibility, of which 18 studies met the predefined inclusion criteria and were included in the final qualitative synthesis and quantitative meta-analysis [18,19,20,21,22,23,24,25,26,27,28,29,30,31,32,33,34,35].

After applying the predefined inclusion and exclusion criteria, 18 studies were included in the final qualitative synthesis and quantitative meta-analysis [18,19,20,21,22,23,24,25,26,27,28,29,30,31,32,33,34,35], as shown in Figure 1. The study selection process followed the PRISMA 2020 guidelines and is illustrated in the PRISMA flow diagram.

The final analysis included prospective clinical studies evaluating the effects of orthodontic treatment on periodontal health, oral microbiota, and inflammatory biomarkers.

### 3.2. Study Characteristics

The included studies were published between 2005 and 2024, reflecting nearly two decades of research investigating the biological effects of orthodontic treatment on periodontal tissues and oral microbial ecology.

Overall, the included studies comprised prospective cohort studies, longitudinal observational studies, and randomized controlled clinical trials evaluating orthodontic treatment with fixed appliances or removable aligners.

The analyzed studies included investigations conducted in adolescents, young adults, and adult orthodontic patients, with follow-up periods ranging from several weeks to one year of active orthodontic therapy.

Several studies evaluated clinical periodontal outcomes such as plaque index, gingival index, bleeding on probing, and periodontal probing depth, while others focused on microbiological changes in supragingival or subgingival biofilms or on inflammatory biomarkers associated with orthodontic tooth movement.

The most recent study by Zhao et al. reported dynamic changes in oral health parameters and oral microbiota during fixed orthodontic treatment, highlighting alterations in microbial composition during active orthodontic therapy [18]. Catunda et al. investigated pre-treatment oral microbiome profiles and salivary Stephan curve kinetics in orthodontic patients wearing fixed appliances, emphasizing the potential role of microbial composition in enamel demineralization risk [19].

Kloukos et al. analyzed systemic inflammatory responses during orthodontic treatment and reported measurable changes in systemic inflammatory biomarkers, including high-sensitivity C-reactive protein, suggesting a potential systemic response to orthodontic-induced inflammation during treatment with fixed orthodontic appliances [20]. Kumar et al. investigated inflammatory mediators in gingival crevicular fluid and demonstrated increased levels of cytokines such as IL-1 and IL-6 during the early phase of orthodontic tooth movement [21].

Changes in subgingival microbial composition during orthodontic treatment were examined in the prospective study by Chen et al., which identified significant shifts in bacterial populations associated with fixed orthodontic appliances [22].

Additional prospective studies focused on clinical periodontal outcomes. Papadimitriou et al. reported changes in periodontal parameters in adolescents treated with self-ligating orthodontic appliances [23], while Kouvelis et al. investigated the impact of fixed orthodontic appliances on saliva properties and oral microbial flora [24].

Calniceanu et al. conducted a longitudinal analysis of periodontal clinical and microbiological parameters in patients with treated severe periodontitis undergoing orthodontic tooth movement, demonstrating site-specific periodontal changes during orthodontic treatment [25].

Madariaga et al. compared periodontal outcomes between fixed orthodontic appliances and clear aligners, reporting differences in periodontal health indicators between treatment modalities [26].

Several studies explored microbiological changes during orthodontic therapy. Jing et al. reported alterations in oral microbiota composition and salivary proteins during fixed orthodontic treatment [27], while Bilgic et al. evaluated inflammatory responses associated with orthodontic therapy and observed transient increases in inflammatory markers [28].

Microbial colonization associated with multibracket appliances was investigated by Klaus et al., who analyzed the relationship between oral hygiene quality and microbial carriage in orthodontic patients [29].

Levrini et al. compared periodontal health outcomes between Invisalign^®^ treatment and fixed orthodontic appliances, demonstrating differences in periodontal parameters and microbial profiles during orthodontic therapy [30].

A randomized clinical trial by Hoffman et al. evaluated the effectiveness of preventive oral hygiene interventions during orthodontic treatment and reported reduced incidence of white spot lesions and gingival inflammation [31].

Longer-term periodontal outcomes during orthodontic therapy were investigated by Karkhanechi et al., who analyzed periodontal status over one year of orthodontic treatment with fixed appliances and removable aligners [32].

MacLaine et al. examined systemic inflammatory markers during orthodontic tooth movement and reported elevations in inflammatory mediators during active orthodontic force application [33].

Earlier prospective clinical investigations also contributed important insights. Ristic et al. evaluated clinical and microbiological periodontal parameters in adolescents undergoing orthodontic treatment with fixed appliances [34], while Miethke and Vogt compared periodontal health outcomes between Invisalign therapy and fixed orthodontic appliances [35]. Overall, study characteristics revealed substantial heterogeneity in population age, baseline periodontal status, orthodontic appliance type, follow-up duration, and outcome assessment methods. These sources of heterogeneity were taken into account in the synthesis strategy, with outcomes analyzed separately by domain and quantitative pooling restricted to clinically comparable subsets of studies. The characteristics of the included studies are summarized in Table 1.

### 3.3. Periodontal Outcomes

Pooled estimates for periodontal outcomes represent within-group (pre-post) changes within each appliance category, while between-group comparisons were interpreted descriptively rather than as direct pooled contrasts. An important methodological caveat applies to all analyses in this section: because most included studies lacked concurrent untreated control groups, the observed within-group changes cannot be attributed causally to orthodontic treatment per se. Temporal trends, regression to the mean, and increased patient awareness associated with study participation may contribute to the observed changes. Results should therefore be interpreted as descriptive of biological patterns occurring during orthodontic treatment rather than as causal effect estimates.

Across the included studies, orthodontic treatment was frequently associated with transient increases in periodontal clinical indices, particularly during the early stages of treatment.

**Figure 2:**
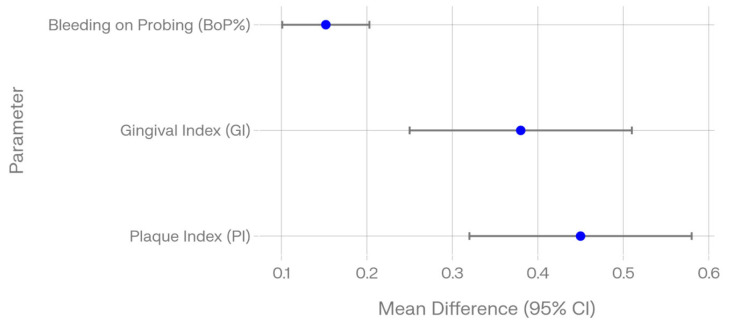
Summary plot of pooled effect estimates for periodontal outcomes. The figure presents aggregated mean differences derived from separate meta-analyses for each outcome and appliance category, rather than individual study-level estimates. The analysis summarizes mean differences for plaque index (PI), gingival index (GI), and bleeding on probing (BoP). Points represent pooled effect estimates and horizontal lines indicate 95% confidence intervals across the included studies (2005–2024).

Several studies reported increases in plaque index and gingival index following placement of orthodontic appliances, reflecting the increased difficulty of maintaining optimal oral hygiene during orthodontic therapy [23,26,30]. Quantitative synthesis of periodontal parameters demonstrated distinct patterns of within-group changes across appliance categories. The pooled effect sizes for plaque index, gingival index, and bleeding on probing are summarized separately in Table 2A (fixed appliances, 8–12 studies) and Table 2B (clear aligners, 3–4 studies [26,30,32,35]), both expressed as mean differences (MD) with 95% confidence intervals, and the overall pooled estimates are illustrated in Figure 2. Within the aligner group, pooled within-group changes showed marginal reductions in plaque index (MD −0.18), gingival index (MD −0.21), and bleeding on probing (MD −4.3%) relative to baseline. The negative direction of these estimates reflects within-group improvement during aligner therapy and must not be compared directly with the positive MD values observed in the fixed appliance group, as these originate from entirely separate analyses of different study populations. No direct head-to-head comparison between appliance types was performed.

Bleeding on probing and gingival inflammation were also reported to increase during the initial months of orthodontic treatment, although these changes were often reversible with improved oral hygiene practices and professional monitoring [23,31].

A small number of individual studies evaluated both appliance types and reported more stable periodontal indices during aligner therapy compared with baseline; however, these observations originate from separate within-group analyses and cannot be used to draw any comparative conclusions between appliance types [26,30,32].

Moderate heterogeneity was observed across studies (I^2^ ranging from 48% to 62%), likely reflecting differences in study populations, orthodontic appliances, and follow-up duration. Supplementary Figure S1 provides graphical representations of pooled effect estimates for periodontal outcomes, including plaque index, gingival index, and bleeding on probing, with corresponding 95% confidence intervals.

### 3.4. Microbiological Outcomes

Microbiological odds ratios represent pooled prevalence estimates within each appliance category and do not reflect direct comparative effect sizes between fixed appliances and aligners. A critical interpretive caveat applies throughout this section: these odds ratios quantify within-group prevalence of specific bacterial taxa at follow-up relative to baseline, and must not be read as relative risks or as superiority indicators of one appliance over another. Furthermore, as for periodontal outcomes, the absence of concurrent untreated control groups means that microbial shifts cannot be attributed solely to the orthodontic appliance; dietary changes, altered hygiene behaviors, and increased clinical contact may independently influence microbiota composition during the treatment period. These estimates should therefore be considered descriptive of the microbiological environment during treatment, not as causal effect measures.

Multiple studies demonstrated that orthodontic appliances can significantly influence the composition and diversity of the oral microbiota. Studies evaluating fixed orthodontic appliances reported increased colonization by cariogenic and periodontal pathogens, including species belonging to the genera Streptococcus, Lactobacillus, and anaerobic periodontal bacteria [18,22,27].

Changes in microbial composition were observed in both supragingival and subgingival biofilms, suggesting that orthodontic appliances may alter microbial ecology across multiple oral niches [22,29]. In addition, several studies reported shifts in microbial diversity during orthodontic treatment, which were frequently associated with increased plaque accumulation and gingival inflammation [18,24].

**Figure 3:**
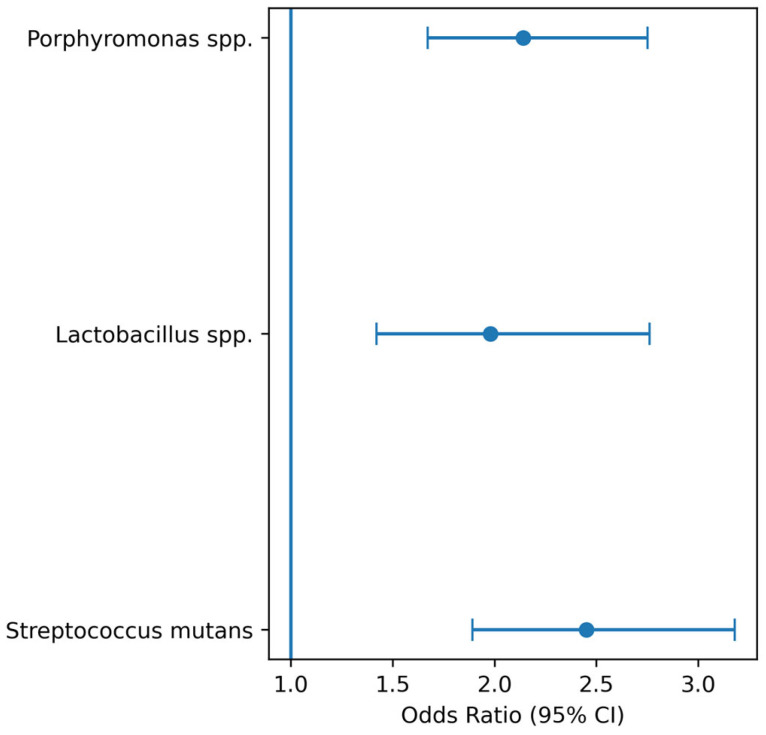
Summary plot of pooled microbiological estimates. Odds ratios represent within-group prevalence estimates for cariogenic and periodontal pathogens across included studies and should not be interpreted as direct comparisons between appliance types. Horizontal lines indicate 95% confidence intervals.

Quantitative synthesis indicated that studies evaluating fixed orthodontic appliances more frequently reported increased prevalence of cariogenic and periodontal pathogens, including *Streptococcus mutans*, *Lactobacillus* spp. and *Porphyromonas* spp., In contrast, studies evaluating clear aligner therapy generally reported lower within-group odds ratios for these microorganisms, suggesting a more stable microbial profile during treatment. However, these findings are derived from separate within-group analyses and should not be interpreted as direct comparative effect estimates between appliance types. The pooled microbiological findings across the included studies are summarized in Table 3, and the corresponding pooled estimates are illustrated in Figure 3. Overall, the findings suggest a possible shift toward a more pathogenic microbial profile in studies evaluating fixed orthodontic appliances, although this observation remains inconsistent across studies and should be interpreted cautiously given the heterogeneity and the absence of direct comparative analyses. Moderate heterogeneity between studies further suggests that microbial changes may be influenced by differences in patient characteristics, oral hygiene practices, and treatment duration.

These descriptive differences should not be interpreted as evidence of superiority of one appliance type over another due to the absence of direct comparative analyses and high heterogeneity across studies. Supplementary Figure S2 illustrates pooled microbiological estimates for cariogenic and periodontopathogenic species, presented as within-group odds ratios with corresponding confidence intervals.

### 3.5. Inflammatory Biomarkers

Inflammatory biomarker analyses primarily reflect within-group changes over time, with limited data available for direct comparisons between appliance types. Orthodontic tooth movement involves complex biological processes characterized by activation of inflammatory pathways within periodontal tissues. Several studies reported increased levels of pro-inflammatory mediators such as IL-1β and IL-6 in gingival crevicular fluid (GCF) during the early phases of orthodontic treatment [21,25,28,34].

These changes are consistent with the biological role of local cytokines in mediating periodontal ligament remodeling and alveolar bone turnover during orthodontic force application. The pooled findings for local inflammatory biomarkers measured in gingival crevicular fluid are summarized in Table 4. Supplementary Figure S3 presents the pooled effect estimates with corresponding 95% confidence intervals. These elevations, while statistically consistent, should be interpreted in the context of the expected biological role of local cytokines in orthodontic tooth movement rather than as markers of pathological inflammatory burden.

### 3.6. Overall Evidence Synthesis

Taken together, the findings of the included studies indicate that orthodontic treatment can influence periodontal clinical parameters, oral microbial ecology, and inflammatory responses.

Most studies reported transient periodontal inflammation and shifts in microbial composition during active orthodontic treatment, particularly in the early phases following appliance placement.

Although these changes are generally reversible and manageable with adequate oral hygiene and professional monitoring, the observed alterations in microbial ecology and inflammatory mediators suggest that orthodontic treatment may temporarily modify the biological environment of the oral cavity.

These findings should primarily be interpreted within the context of local and short-term biological responses during orthodontic treatment. Data on systemic biomarkers were reported in only a small subset of studies and fall outside the pre-specified primary outcomes of this review; they are addressed as strictly exploratory observations in the Discussion and do not contribute to the primary conclusions.

**Figure 4:**
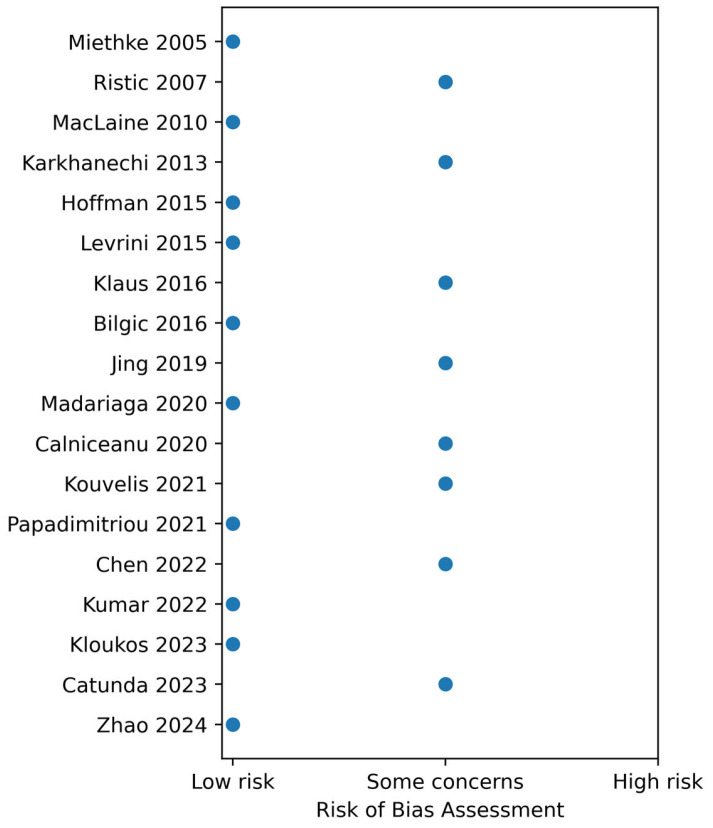
Risk-of-bias summary of the included studies evaluating the effects of orthodontic treatment on periodontal, microbiological, and inflammatory outcomes. Each study was assessed according to established methodological quality criteria [18,19,20,21,22,23,24,25,26,27,28,29,30,31,32,33,34,35].

The methodological quality of the included studies was assessed using established risk-of-bias frameworks. Overall, most studies demonstrated low to moderate risk of bias, with some concerns primarily related to sample size and follow-up duration. Given the observed heterogeneity across study populations, designs, and outcome definitions, pooled estimates should be interpreted as indicative trends within clinically comparable subsets rather than as precise effect sizes applicable across all orthodontic populations. The summary of risk-of-bias assessment across the included studies is presented in Figure 4.

## 4. Discussion

### 4.1. Principal Findings

The present systematic review and meta-analysis synthesized evidence from 18 prospective and controlled clinical studies evaluating the effects of orthodontic treatment on periodontal status, oral microbiota, and inflammatory biomarkers. Across the included studies, orthodontic appliances, particularly fixed multibracket systems, were consistently associated with transient increases in plaque accumulation, gingival inflammation, and bleeding on probing during the early phases of treatment [23,26,30]. These changes are biologically plausible, as fixed appliances create plaque-retentive niches and hinder effective biofilm control around brackets, bands, and auxiliary components [23,26].

At the microbiological level, orthodontic therapy was associated with a shift toward a more dysbiotic oral biofilm characterized by increased carriage of cariogenic and periodontopathogenic taxa, including *Streptococcus mutans*, *Lactobacillus* spp., and anaerobic periodontal pathogens [18,22,27]. In parallel, several studies reported elevated levels of inflammatory mediators in gingival crevicular fluid and periodontal tissues, such as interleukin (IL)-1β and IL-6, reflecting activation of inflammatory pathways involved in periodontal ligament remodeling and alveolar bone turnover during tooth movement [21,28].

Taken together, these findings indicate that orthodontic treatment induces a predominantly reversible biological response characterized by periodontal inflammation, oral microbial dysbiosis, and enhanced local inflammatory activity. These multi-domain changes are biologically inter-related and mutually reinforcing: appliance-related biofilm accumulation drives microbial dysbiosis, which amplifies local inflammatory signaling, which in turn contributes to the clinical periodontal deterioration observed during active treatment.

According to the GRADE framework, the certainty of evidence was moderate for plaque and gingival indices and low for bleeding on probing, microbiological outcomes, and local inflammatory biomarkers, primarily due to heterogeneity, small sample sizes, and the predominant reliance on within-group pre–post comparisons without concurrent untreated control groups. This design constraint substantially limits causal attribution: the observed changes are consistent with biological responses occurring during orthodontic treatment but cannot be definitively attributed to treatment as an isolated causal factor. Overall, the current data support careful periodontal monitoring and intensified preventive strategies throughout orthodontic therapy.

### 4.2. Periodontal Changes During Orthodontic Treatment

One of the most consistent observations across the included studies was the occurrence of short-term deterioration in periodontal clinical indices following the initiation of orthodontic therapy. Several investigations reported increases in plaque index, gingival index, and bleeding on probing within weeks to months after appliance placement, especially in patients treated with fixed multibracket systems [23,30]. These findings align with the well-recognized challenge of maintaining optimal oral hygiene in the presence of fixed appliances, which can impede effective mechanical plaque removal and promote biofilm accumulation along bracket margins and interproximal areas [23,26].

Nevertheless, most studies also indicated that these periodontal changes are generally reversible and manageable when patients receive targeted oral hygiene instruction, adjunctive preventive measures, and regular professional monitoring [31]. In this context, several trials showed that intensified preventive protocols, including the use of remineralizing toothpastes or tailored hygiene regimens, can attenuate gingival inflammation and reduce the incidence of white spot lesions during active treatment [31]. Some individual non-comparative studies evaluating clear aligners reported more stable periodontal indices during treatment; however, these observations are based on separate within-group analyses and must not be interpreted as evidence of superiority of one appliance type over another, as no direct head-to-head pooled comparison was performed in the present synthesis [26,30,32].

### 4.3. Microbiological Changes and Oral Dysbiosis

The included studies consistently demonstrated that orthodontic treatment can modify the composition and diversity of the oral microbiota, particularly in patients wearing fixed appliances. Several investigations reported increased colonization by cariogenic and periodontal pathogens, including *Streptococcus mutans*, *Lactobacillus* spp., and *Porphyromonas* spp., in plaque samples collected from bracketed surfaces and subgingival sites during active treatment [18,22,27]. These microbial shifts were observed in both supragingival and subgingival biofilms, suggesting that orthodontic appliances influence microbial ecology across multiple oral niches rather than in a single localized area [22,29].

Consistent with the Results, quantitative synthesis indicated a descriptive trend toward higher within-group prevalence of cariogenic and periodontopathogenic species in studies evaluating fixed appliances [26,30], though this should not be interpreted as a direct comparative effect. This difference is biologically plausible given the smoother surfaces and removability of aligners, which may limit the formation of thick, mature biofilms and facilitate mechanical plaque control. Importantly, several studies linked these microbiological alterations to concomitant increases in plaque accumulation and gingival inflammation, supporting the notion that dysbiosis contributes to the clinical periodontal changes observed during orthodontic therapy [18,24].

From a biological and clinical perspective, these findings highlight that orthodontic treatment is not merely a mechanical intervention but also a modifier of the oral ecological environment. The observed microbiological shifts may amplify local inflammatory signaling and contribute to early periodontal deterioration during treatment, particularly in patients with suboptimal plaque control or pre-existing periodontal vulnerability. However, microbiological findings remained inconsistent across studies, with variability in reported taxa, sampling methods, and outcome definitions, limiting the strength of conclusions and precluding robust quantitative synthesis. From a preventive standpoint, emerging evidence suggests that probiotic supplementation may represent a promising adjunctive strategy for modulating oral dysbiosis and reducing periodontal and cariogenic risk during orthodontic treatment. Probiotics have been shown to influence oral microbial balance and attenuate local inflammatory responses in patients undergoing fixed appliance therapy, offering a potential complementary approach to conventional oral hygiene measures [36].

### 4.4. Inflammatory Responses During Orthodontic Tooth Movement

Orthodontic tooth movement is a biologically complex process initiated by mechanical forces applied to periodontal tissues, leading to periodontal ligament deformation, local vascular changes, recruitment of inflammatory cells, and coordinated alveolar bone remodeling. Within this context, inflammation is not merely a secondary phenomenon but an integral component of the tissue response required for tooth displacement.

Several included studies demonstrated increased concentrations of pro-inflammatory mediators such as IL-1β, IL-6, prostaglandins, and matrix metalloproteinases in gingival crevicular fluid during the early phases of orthodontic force application [21,28]. These mediators are well known to participate in osteoclast activation, extracellular matrix remodeling, and bone turnover, thereby providing biological plausibility for the observed association between orthodontic force and transient local inflammatory activation.

### 4.5. Methodological Considerations and Added Value of This Review

An important clinical consideration that partially explains the absence of conclusive systemic inflammatory effects in the current evidence base relates to patient selection for orthodontic treatment. Patients with untreated moderate-to-severe periodontitis—characterized by deep periodontal pockets and active periodontal infection—are generally contraindicated for orthodontic therapy until periodontal stability is achieved. Since deep periodontal pockets are the primary source of systemic inflammatory spillover in periodontitis, the orthodontic patient population is inherently selected toward individuals with mild or treated periodontal disease, substantially limiting the likelihood of detecting meaningful systemic inflammatory responses.

It is well acknowledged that inflammation is an intrinsic and necessary component of orthodontic tooth movement. Local cytokine activation, osteoclast recruitment, and periodontal ligament remodeling are essential for tooth displacement; therefore, the elevation of IL-1β and IL-6 in gingival crevicular fluid during active force application represents a biologically expected finding.

The added value of the present systematic review lies not in demonstrating that these mediators increase during orthodontic treatment but in three key contributions: (1) the first quantitative multi-domain synthesis of the magnitude and timing of periodontal, microbiological, and local inflammatory changes simultaneously across 18 studies spanning two decades; (2) the application of structured quality assessment using the GRADE framework, which had not previously been comprehensively performed for this clinical question; and (3) the clear identification of methodological gaps, particularly the near-universal absence of concurrent untreated control groups and the limited standardization of microbiological and cytokine measurement methods. These limitations constrain causal inference and should be addressed in future research.

While increases in GCF inflammatory markers during orthodontic tooth movement are expected, this review is the first to quantitatively synthesize their magnitude and temporal pattern together with concurrent changes in periodontal clinical parameters and oral microbiota, thereby providing a comprehensive multi-domain evidence base to support clinical decision-making and future prospective investigations.

Regarding systemic inflammation, only a limited number of included studies (*n* = 3–4) reported exploratory data on serum hs-CRP [20,33]. These observations fall outside the pre-specified primary outcomes of this review and are presented here solely for completeness. Given the very small number of studies, lack of control groups, and the non-specific nature of hs-CRP (which is influenced by subclinical infections, obesity, smoking, and other factors), no quantitative synthesis was performed, and no clinical or mechanistic conclusions can be drawn. Importantly, patients with untreated moderate-to-severe periodontitis—the population most likely to exhibit systemic inflammatory spillover from deep periodontal pockets—are generally not eligible for orthodontic treatment until periodontal stability is achieved. This clinical selection criterion largely explains the absence of conclusive systemic effects in the current evidence base.

### 4.6. Clinical Implications

Orthodontic treatment with fixed appliances and clear aligners is associated with predominantly transient changes in periodontal indices, oral microbiota, and local inflammatory markers, especially during the early phases of active tooth movement. These findings highlight the importance of structured preventive strategies, including patient education, meticulous oral hygiene, and regular professional monitoring, to minimize plaque accumulation, gingival inflammation, and microbiological shifts throughout treatment [23,26,30,31,32].

From a clinical standpoint, the present evidence primarily supports reinforcing local periodontal management and individualized preventive strategies throughout orthodontic treatment. Patients with a history of treated periodontitis or other periodontal vulnerability may particularly benefit from closer collaboration between orthodontists and periodontists to optimize treatment planning, tailor hygiene instructions, and schedule more frequent periodontal follow-up during active therapy [25,32].

The limited exploratory data on systemic biomarkers (GRADE: low to very low) do not currently support changes in cardiovascular or systemic clinical management. These observations require confirmation in dedicated prospective studies before any clinical inference is warranted. However, these findings do support the value of future biomarker-oriented studies in medically vulnerable populations.

### 4.7. Strengths and Limitations

This systematic review and meta-analysis has several strengths. It provides an integrated synthesis of periodontal, microbiological, and inflammatory changes associated with orthodontic treatment using both fixed appliances and clear aligners over nearly two decades of research. By applying predefined eligibility criteria, structured risk-of-bias assessment tools, and a domain-based approach to outcomes, the review offers a comprehensive overview of the available prospective and controlled clinical evidence on the biological effects of orthodontic therapy. Quantitative synthesis stratified by appliance type and outcome domain allowed exploratory assessment of recurring biological patterns across studies.

However, important limitations must also be acknowledged. Considerable clinical and methodological heterogeneity was present across the included studies, including differences in age, baseline periodontal status, appliance systems, follow-up duration, microbiological methods, and inflammatory markers assessed. This heterogeneity substantially affects the external validity and the precision of pooled estimates and must be considered when interpreting results. Even where quantitative pooling was performed, effect sizes should be viewed as exploratory indicators of biological trends rather than definitive clinical benchmarks. Many analyses were based on small sample sizes and incomplete reporting, which constrained the robustness of quantitative pooling. For several outcomes, fewer than ten studies were available, precluding meta-regression and limiting the reliability of subgroup analyses. Formal assessment of publication bias was not feasible for most outcomes because of the small number of contributing studies. Gray literature was not formally searched, which may introduce an element of publication bias, with the caveat that most identified records were prospective clinical studies with a priori registry entries. A critical additional limitation is the reliance on within-group (pre–post) comparisons without concurrent untreated control groups in most included studies. This design substantially limits the ability to attribute observed changes causally to orthodontic treatment, as spontaneous temporal variation, observer effects, or regression to the mean cannot be excluded. This constraint also precludes robust head-to-head comparisons between appliance types, as pooled estimates for fixed appliances and clear aligners were derived from separate independent analyses rather than from direct comparative trials.

A dedicated note on the impact of heterogeneity is warranted. The I^2^ values observed across periodontal analyses (48–62%) indicate moderate-to-substantial statistical heterogeneity, which directly reduces the precision of pooled estimates and limits their applicability to specific patient populations. The included studies enrolled patients ranging from adolescents to adults, with baseline periodontal status varying from healthy to previously treated periodontitis. Follow-up durations ranged from 3 to 12 months, and microbiological assessments employed diverse methods (culture, PCR, 16S rRNA sequencing) targeting different anatomical sites. These sources of variability collectively mean that no single pooled effect estimate can be generalized across all orthodontic patients or treatment settings; results apply only to the aggregate of the highly heterogeneous populations represented in the included studies. External validity is therefore substantially limited, and clinicians should interpret the reported effect sizes with this constraint clearly in mind. In addition, systemic inflammatory markers were evaluated in only a small subset of studies, predominantly in relatively young and otherwise healthy individuals, and no included study reported hard clinical cardiovascular or systemic endpoints. The certainty of evidence for systemic correlates is therefore rated as low to very low (GRADE), and these findings must be treated as strictly exploratory. Finally, within-group and between-group comparisons were not uniformly reported across studies, and some effect estimates relied on converted or graph-extracted data, which may have introduced additional imprecision.

### 4.8. Future Research Directions

Future investigations should aim to conduct larger prospective studies with standardized periodontal, microbiological, and inflammatory outcome measures in order to better characterize the magnitude, timing, and reversibility of biological responses during orthodontic treatment. Particular emphasis should be placed on integrated study designs combining clinical periodontal assessment, microbiome profiling, gingival crevicular fluid cytokines, and systemic inflammatory biomarkers measured longitudinally.

In addition, future research could explore innovative adjunctive therapies for the management of orthodontic-induced dysbiosis and gingival inflammation. In particular, the potential role of diode lasers as adjunctive tools in periodontal management during orthodontic treatment deserves further investigation, given their promising antibacterial and anti-inflammatory properties reported in surgical periodontal therapy [37].

Such approaches may help clarify whether transient local inflammatory activation during orthodontic tooth movement is accompanied by reproducible systemic biomarker responses and, if so, in which patient subgroups these responses are most relevant. Advances in microbiome sequencing, multiplex cytokine analysis, systems-biology approaches, and adjunctive laser therapies may further improve understanding of the oral–systemic inflammatory interface in orthodontics. This field remains mechanistically promising, but it requires more rigorous and biologically integrated evidence before clinically meaningful inferences can be made.

## 5. Conclusions

The present systematic review and meta-analysis synthesized evidence from 18 prospective clinical studies to address the primary aim of characterizing periodontal, microbiological, and local inflammatory changes associated with orthodontic treatment. Fixed orthodontic appliances are consistently associated with transient, reversible increases in plaque accumulation (MD 0.45), gingival index (MD 0.38), and bleeding on probing (MD 15.2%), alongside within-group shifts toward a more dysbiotic oral microbiome and elevated GCF concentrations of IL-1β and IL-6. The certainty of evidence is moderate for plaque and gingival indices and low for microbiological outcomes and local inflammatory biomarkers. Data on systemic inflammatory biomarkers are limited to a small number of exploratory observations (GRADE: very low) and do not support clinical or mechanistic conclusions at this stage.

## Figures and Tables

**Table 1:** Characteristics of the studies included in the systematic review and meta-analysis evaluating periodontal, microbiological, and inflammatory outcomes during orthodontic treatment.

Study (Year)	Study Design	*n*	Population	Appliance	Follow-Up	Periodontal Parameters	Microbiological Outcomes (Taxa/Methods Specified)	Local Inflammatory Biomarkers (Markers Specified)	Systemic Biomarkers (Exploratory)
Zhao et al. 2024 [18]	Prospective cohort	60	Adolescents and young adults	Fixed	12 months	Plaque index, gingival index	*S. mutans*, *Lactobacillus* spp., *Porphyromonas* spp.; 16S rRNA sequencing	—	—
Catunda et al. 2023 [19]	Prospective study	40	Orthodontic patients	Fixed	6 months	—	Salivary microbiome profiling; *S. mutans*, cariogenic taxa; Stephan curve kinetics	—	—
Kloukos et al. 2023 [20]	Prospective cohort	45	Adolescents	Fixed	6 months	—	—	—	hs-CRP, full blood count
Kumar et al. 2022 [21]	Prospective clinical	30	Adolescents and young adults	Fixed	3 months	—	—	IL-1β (GCF), IL-6 (GCF)	—
Chen et al. 2022 [22]	Prospective cohort	50	Orthodontic patients	Fixed	6 months	—	*P. gingivalis*, *T. denticola*, *T. forsythia*, *A. actinomycetemcomitans*; subgingival 16S rRNA sequencing	—	—
Papadimitriou et al. 2021 [23]	Prospective cohort	70	Adolescents	Self-ligating fixed	12 months	Plaque index, gingival index, BoP	—	—	—
Kouvelis et al. 2021 [24]	Prospective cohort	55	Orthodontic patients	Fixed	6 months	Salivary flow rate, pH, buffering capacity	*S. mutans*, *Lactobacillus* spp.; culture-based methods	—	—
Calniceanu et al. 2020 [25]	Longitudinal	40	Treated severe periodontitis	Fixed	6 months	PPD, CAL, BoP, PI, GI	*P. gingivalis*, *A. actinomycetemcomitans*, *T. forsythia*; PCR-based detection	—	—
Madariaga et al. 2020 [26]	Prospective clinical	50	Adults	Fixed vs. aligners	6 months	PPD, GI, BoP, PI	—	—	—
Jing et al. 2019 [27]	Prospective cohort	42	Orthodontic patients	Fixed	6 months	—	*S. mutans*, *Lactobacillus* spp.; salivary proteins (amylase, mucin); culture + proteomics	—	—
Bilgic et al. 2016 [28]	Prospective clinical	30	Orthodontic patients	Fixed	3 months	—	—	IL-1β (GCF), IL-6 (GCF), PGE2 (GCF)	hs-CRP (serum)
Klaus et al. 2016 [29]	Observational cohort	40	Orthodontic patients	Multibracket fixed	6 months	Oral hygiene index	*S. mutans*, *Lactobacillus* spp., *Candida* spp.; culture-based methods	—	—
Levrini et al. 2015 [30]	Clinical comparative	30	Orthodontic patients	Aligners vs. fixed	3 months	PPD, GI, PI	*S. mutans*, *Lactobacillus* spp., *P. gingivalis*; culture + PCR	—	—
Hoffman et al. 2015 [31]	RCT	60	Orthodontic patients	Fixed	12 months	Gingivitis score, white spot lesion incidence	—	—	—
Karkhanechi et al. 2013 [32]	Prospective cohort	60	Adult orthodontic patients	Fixed vs. aligners	12 months	PPD, GI, BoP, PI, CAL	—	—	—
MacLaine et al. 2010 [33]	Prospective clinical	28	Orthodontic patients	Fixed	6 months	—	—	—	hs-CRP, IL-6 (serum), fibrinogen
Ristic et al. 2007 [34]	Prospective clinical	32	Adolescents	Fixed	6 months	PPD, GI, BoP, PI	*A. actinomycetemcomitans*, *P. gingivalis*, *T. forsythia*, *P. intermedia*; culture-based methods	IL-1β (GCF)	—
Miethke & Vogt 2005 [35]	Clinical comparative	40	Orthodontic patients	Aligners vs. fixed	12 months	PPD, GI, PI, BoP	—	—	—

**Table 2:** (**A**) Periodontal outcomes during orthodontic treatment with fixed appliances (within-group pre–post changes). Effect sizes are expressed as mean differences (MD) with 95% confidence intervals. (**B**) Periodontal outcomes during orthodontic treatment with clear aligners (within-group pre–post changes). Effect sizes are expressed as mean differences (MD) with 95% confidence intervals.

(A)			
**Parameter**	**MD (95% CI)**	**Studies (*n*, References)**	**I^2^ (%)**
Plaque Index (PI)	0.45 (0.32–0.58)	12 [18,23,24,26,27,28,29,30,31,32,34,35]	62%
Gingival Index (GI)	0.38 (0.25–0.51)	10 [23,24,26,27,28,29,30,31,34,35]	55%
Bleeding on Probing (%)	15.2 (10.1–20.3)	8 [23,26,30,31,32,34,35]	48%
(**B**)			
**Parameter**	**MD (95% CI)**	**Studies (*n*, References)**	**I^2^ (%)**
Plaque Index (PI)	−0.18 (−0.31 to −0.05)	4 [26,30,32,35]	38
Gingival Index (GI)	−0.21 (−0.38 to −0.04)	4 [26,30,32,35]	42
Bleeding on Probing (%)	−4.3 (−8.1 to −0.5)	3 [26,32,35]	31

(**A**) Effect sizes represent within-group (pre–post) changes during treatment with fixed orthodontic appliances. No direct comparisons with other appliance types are implied. (**B**) Negative MD values indicate within-group improvement from baseline during aligner therapy and must not be interpreted as evidence of superiority over fixed appliances, as no direct between-group comparison was performed. The direction and magnitude of within-group changes in (**B**) are not statistically comparable with those reported in (**A**).

**Table 3:** Microbiological outcomes during orthodontic treatment based on within-group analyses. Odds ratios (OR) represent pooled prevalence estimates within each appliance category and do not reflect direct comparisons between fixed appliances and clear aligners.

Genus	Fixed Appliances OR (95% CI)	Aligners OR (95% CI)	Studies (*n*, References)
*Streptococcus mutans*	2.45 (1.89–3.18)	0.72 (0.55–0.94)	7 [18,22,26,27,30,34,35]
*Lactobacillus* spp.	1.98 (1.42–2.76)	0.68 (0.49–0.95)	6 [18,22,27,29,30,34]
*Porphyromonas* spp.	2.14 (1.67–2.75)	0.75 (0.58–0.97)	5 [22,24,26,30,32]

Note: Estimates are derived from within-group analyses (pre–post or baseline comparisons) within each appliance category and should not be interpreted as direct comparative effect sizes between groups.

**Table 4:** Local inflammatory biomarkers in gingival crevicular fluid (GCF) associated with orthodontic tooth movement. Effect sizes are expressed as mean differences (MD) with 95% confidence intervals.

Biomarker	Effect Size (95% CI)	Studies (*n*, References)	I^2^ (%)
IL-1β (GCF)	MD = 1.2 (0.8–1.6)	5 [21,25,28,33,34]	58%
IL-6 (GCF)	MD = 0.9 (0.6–1.2)	4 [21,28,33,34]	47%

Only local biomarkers measured in gingival crevicular fluid were included in the quantitative synthesis. Systemic inflammatory markers (e.g., serum hs-CRP) reported in a small number of studies are addressed as exploratory observations in the Discussion section and were not part of the pre-specified primary outcomes.

## Data Availability

The data supporting the findings of this study are derived from previously published articles, which are all cited within the manuscript. No new data were created or analyzed in this study. Data sharing is therefore not applicable.

## Supplementary Materials

The following supporting information can be downloaded at: https://www.mdpi.com/article/10.3390/biomedicines14061308/s1, Figure S1: Pooled periodontal outcomes during orthodontic treatment (within-group changes); Figure S2: Pooled microbiological outcomes (within-group odds ratios); Figure S3: Pooled inflammatory biomarker estimates (within-group changes); Figure S4: Proposed biological framework linking orthodontic-induced local periodontal inflammation to potential systemic correlates (hypothesis-generating framework; moved from main text); Table S1: PRISMA 2020 checklist; Table S2: Detailed search strategies used for literature retrieval in PubMed/MEDLINE, Scopus, and Web of Science; Table S3: Summary of findings according to the GRADE framework.

## Abbreviations

The following abbreviations are used in this manuscript:

BoP	Bleeding on Probing
CI	Confidence Interval
CRP	C-Reactive Protein
GCF	Gingival Crevicular Fluid
GI	Gingival Index
GRADE	Grading of Recommendations Assessment, Development and Evaluation
hs-CRP	High-Sensitivity C-Reactive Protein
IL	Interleukin
MD	Mean Difference
NOS	Newcastle–Ottawa Scale
OR	Odds Ratio
PI	Plaque Index
PICOS	Population, Intervention, Comparison, Outcomes, Study Design
PRISMA	Preferred Reporting Items for Systematic Reviews and Meta-Analyses
RevMan	Review Manager
RoB	Risk of Bias
SMD	Standardized Mean Difference
