# Supplementary material for: Orthodontic Treatment-Induced Periodontal, Microbiological, and Local Inflammatory Changes: A Systematic Review and Meta-Analysis
Source: Biomedicines. 2026 Jun 9;14(6):1308. doi: 10.3390/biomedicines14061308 (PMC13297047; doi:10.3390/biomedicines14061308)
Supplement: Supplementary file 1 [file biomedicines-14-01308-s001.zip › biomedicines-4292142-supplementary.pdf]

Supplementary Materials

Supplementary Table S1: PRISMA 2020 checklist.

Section and Topic	Item #	Checklist item	Location where item is reported
**TITLE**			
Title	1	Identify the report as a systematic review.	Title page "Orthodontic Treatment–Induced Periodontal, Microbiological, and Local Inflammatory Changes: A Systematic Review and Meta-Analysis"
**ABSTRACT**			
Abstract	2	See the PRISMA 2020 for Abstracts checklist.	Page 1 – Abstract section.
**INTRODUCTION**			
Rationale	3	Describe the rationale for the review in the context of existing knowledge.	Introduction, paragraphs 1–4.
Objectives	4	Provide an explicit statement of the objective(s) or question(s) the review addresses.	End of Introduction.
**METHODS**			
Eligibility criteria	5	Specify the inclusion and exclusion criteria for the review and how studies were grouped for the syntheses.	Section 2.2 Eligibility Criteria (PICOS Framework)
Information sources	6	Specify all databases, registers, websites, organisations, reference lists and other sources searched or consulted to identify studies. Specify the date when each source was last searched or consulted.	Section 2.3 Information Sources and Search Strategy. Databases:<br><br>Section 2.3 Information Sources and Search Strategy. Databases: PubMed/MEDLINE, Scopus, Web of Science. Reference lists of all included studies

Section and Topic	Item #	Checklist item	Location where item is reported
			and relevant systematic reviews were also manually screened. Grey literature was not formally searched (rationale and potential bias discussed in Section 4.7). No language restrictions were applied.
Search strategy	7	Present the full search strategies for all databases, registers and websites, including any filters and limits used.	Section 2.3 Search Strategy. Complete search strategies are provided in Supplementary Table S2.
Selection process	8	Specify the methods used to decide whether a study met the inclusion criteria of the review, including how many reviewers screened each record and each report retrieved, whether they worked independently, and if applicable, details of automation tools used in the process.	Section 2.4 Study Selection. Titles and abstracts were screened independently by two reviewers, followed by full-text assessment according to predefined inclusion criteria.
Data collection process	9	Specify the methods used to collect data from reports, including how many reviewers collected data from each report, whether they worked independently, any processes for obtaining or confirming data from study investigators, and if applicable, details of automation tools used in the process.	Section 2.5 Data Extraction. Data were extracted using a standardized extraction template and cross-checked for accuracy.

Section and Topic	Item #	Checklist item	Location where item is reported
Data items	10a	List and define all outcomes for which data were sought. Specify whether all results that were compatible with each outcome domain in each study were sought (e.g. for all measures, time points, analyses), and if not, the methods used to decide which results to collect.	Section 2.2 Eligibility Criteria – Outcomes<br>Outcomes include:<br>• plaque index<br>• gingival index<br>• bleeding on probing<br>• microbiological outcomes<br>• local inflammatory biomarkers (IL-1β, IL-6); exploratory systemic biomarkers (hs-CRP)
	10b	List and define all other variables for which data were sought (e.g. participant and intervention characteristics, funding sources). Describe any assumptions made about any missing or unclear information.	Section 2.5 Data Extraction<br>Variables extracted:<br>• study design<br>• sample size<br>• population characteristics<br>• orthodontic appliance type<br>• follow-up duration<br>• periodontal parameters<br>• microbiological outcomes<br>• inflammatory biomarkers
Study risk of bias assessment	11	Specify the methods used to assess risk of bias in the included studies, including details of the tool(s) used, how many reviewers assessed each study and whether they worked independently, and if applicable, details of automation tools used in the process.	Section 2.6 Risk of Bias Assessment<br>Tools used:<br>• RoB 2 for randomized trials<br>• Newcastle–Ottawa Scale (NOS) for

Section and Topic	Item #	Checklist item	Location where item is reported
			observational studies
Effect measures	12	Specify for each outcome the effect measure(s) (e.g. risk ratio, mean difference) used in the synthesis or presentation of results.	Location:<br>Section 2.8 Statistical Analysis<br>Effect measures:<br>• Mean Difference (MD)<br>• Standardized Mean Difference (SMD)<br>• Odds Ratio (OR)
Synthesis methods	13a	Describe the processes used to decide which studies were eligible for each synthesis (e.g. tabulating the study intervention characteristics and comparing against the planned groups for each synthesis (item #5)).	Section 2.8 Statistical Analysis
	13b	Describe any methods required to prepare the data for presentation or synthesis, such as handling of missing summary statistics, or data conversions.	Section 2.8 Statistical Analysis
	13c	Describe any methods used to tabulate or visually display results of individual studies and syntheses.	Location:<br>Results section:<br>• Tables 1–4<br>• Figures 1–4
	13d	Describe any methods used to synthesize results and provide a rationale for the choice(s). If meta-analysis was performed, describe the model(s), method(s) to identify the presence and extent of statistical heterogeneity, and software package(s) used.	Section 2.8 Statistical Analysis<br>Meta-analysis model:<br>• Fixed-effect or Random-effects (DerSimonian–Laird)<br>• Heterogeneity assessed with I^2 statistic<br>• Software: Review Manager (RevMan) 5.4

Section and Topic	Item #	Checklist item	Location where item is reported
	13e	Describe any methods used to explore possible causes of heterogeneity among study results (e.g. subgroup analysis, meta-regression).	Section 2.8 Statistical Analysis<br>Subgroup analyses based on:<br>• appliance type<br>• outcome category<br>• treatment duration
	13f	Describe any sensitivity analyses conducted to assess robustness of the synthesized results.	Section 2.8 Statistical Analysis<br>Sensitivity analyses not performed due to limited number of studies.
Reporting bias assessment	14	Describe any methods used to assess risk of bias due to missing results in a synthesis (arising from reporting biases).	Section 4.7 Strengths and Limitations
Certainty assessment	15	Describe any methods used to assess certainty (or confidence) in the body of evidence for an outcome.	Section 2.7 Certainty of Evidence<br>GRADE framework.<br>Results summarized in Supplementary Table S3.
**RESULTS**			
Study selection	16a	Describe the results of the search and selection process, from the number of records identified in the search to the number of studies included in the review, ideally using a flow diagram.	Section 3.1 Study Selection<br>PRISMA diagram: Figure 1<br>Numbers:<br>• Records identified: 1248<br>• After duplicates: 812<br>• Full texts assessed: 142<br>• Included studies: 18
	16b	Cite studies that might appear to meet the inclusion criteria, but which were excluded, and explain why	Section 3.1 Study Selection

Section and Topic	Item #	Checklist item	Location where item is reported
		they were excluded.	Reasons described in text.
Study characteristics	17	Cite each included study and present its characteristics.	Section 3.2 Study Characteristics<br>Presented in Table 1.
Risk of bias in studies	18	Present assessments of risk of bias for each included study.	Section 3.6 Overall Evidence Synthesis<br>Presented in Figure 4.
Results of individual studies	19	For all outcomes, present, for each study: (a) summary statistics for each group (where appropriate) and (b) an effect estimate and its precision (e.g. confidence/credible interval), ideally using structured tables or plots.	Tables:<br>• Table 1<br>• Table 2<br>• Table 3<br>• Table 4
Results of syntheses	20a	For each synthesis, briefly summarise the characteristics and risk of bias among contributing studies.	Section 3.6 Overall Evidence Synthesis
	20b	Present results of all statistical syntheses conducted. If meta-analysis was done, present for each the summary estimate and its precision (e.g. confidence/credible interval) and measures of statistical heterogeneity. If comparing groups, describe the direction of the effect.	Tables 2–4, Figures 2–4
	20c	Present results of all investigations of possible causes of heterogeneity among study results.	Results sections 3.3–3.5<br>I^2 values reported.
	20d	Present results of all sensitivity analyses conducted to assess the robustness of the synthesized results.	Not performed due to limited number of studies.
Reporting biases	21	Present assessments of risk of bias due to missing results (arising from reporting biases) for each synthesis assessed.	Section 4.7 Strengths and Limitations
Certainty of evidence	22	Present assessments of certainty (or confidence) in the body of evidence for each outcome assessed.	Supplementary Table S3 (GRADE).
**DISCUSSION**			
Discussion	23a	Provide a general interpretation of the results in the context of other evidence.	Section 4.1 Principal Findings

Section and Topic	Item #	Checklist item	Location where item is reported
	23b	Discuss any limitations of the evidence included in the review.	Section 4.7 Strengths and Limitations
	23c	Discuss any limitations of the review processes used.	Section 4.7 Strengths and Limitations
	23d	Discuss implications of the results for practice, policy, and future research.	Sections:<br>• 4.6 Clinical Implications<br>• 4.8 Future Research Directions
**OTHER INFORMATION**			
Registration and protocol	24a	Provide registration information for the review, including register name and registration number, or state that the review was not registered.	Section 2.1 Study Design and Reporting Standards. PROSPERO: CRD420261336117
	24b	Indicate where the review protocol can be accessed, or state that a protocol was not prepared.	Registered on PROSPERO.
	24c	Describe and explain any amendments to information provided at registration or in the protocol.	One post-hoc amendment: exploratory narrative analysis of systemic inflammatory biomarkers was added based on reporting in included studies. Systemic outcomes were not pre-specified as primary outcomes in the PROSPERO protocol. This amendment is transparently reported in Section 2.1 and interpreted strictly as hypothesis-generating

Section and Topic	Item #	Checklist item	Location where item is reported
			throughout the manuscript.
Support	25	Describe sources of financial or non-financial support for the review, and the role of the funders or sponsors in the review.	No external funding was received for this study.
Competing interests	26	Declare any competing interests of review authors.	Conflicts of Interest section. None declared.
Availability of data, code and other materials	27	Report which of the following are publicly available and where they can be found: template data collection forms; data extracted from included studies; data used for all analyses; analytic code; any other materials used in the review.	Data Availability Statement: Data supporting the findings of this study are available from the corresponding author upon reasonable request.

Supplementary Table S2. Detailed search strategies used for literature retrieval in PubMed/MEDLINE, Scopus, and Web of Science.

Database	Search query	Filters
PubMed/MEDLINE	("orthodontic treatment" OR "orthodontic appliances" OR "clear aligners") AND ("oral microbiota" OR "oral microbiome") AND ("cytokines" OR "inflammatory biomarkers" OR "C-reactive protein")	Humans; No date restriction
Scopus	TITLE-ABS-KEY ("orthodontic treatment" OR "orthodontic appliances" OR "clear aligners") AND TITLE-ABS-KEY ("oral microbiota" OR "oral microbiome") AND TITLE-ABS-KEY ("cytokines" OR "inflammatory biomarkers" OR "C-reactive protein")	Article
Web of Science	TS=("orthodontic treatment" OR "orthodontic appliances" OR "clear aligners") AND TS=("oral microbiota" OR "oral microbiome") AND TS=("cytokines" OR "inflammatory biomarkers" OR "C-reactive protein")	Article

Supplementary Table S3. Summary of findings according to the GRADE framework for periodontal, microbiological, and inflammatory outcomes during orthodontic treatment.

**Outcome**	**Number of studies**	**Effect estimate**	**Certainty of evidence**	**Main reasons for downgrading**
Plaque Index	12 [18,23,24,26–32,34,35]	MD 0.45	Moderate	Heterogeneity between studies
Gingival Index	10 [23,24,26–31,34,35]	MD 0.38	Moderate	Clinical heterogeneity
Bleeding on probing	8 [23,26,30–32,34,35]	MD 15.2	Low	Small number of studies
IL-6	4 [21,28,33,34]	MD 0.9	Low	Small sample size and heterogeneity
hs-CRP	3 [20,28,33]	SMD 0.42	Low	Very limited number of studies (n=3); no concurrent controls; heterogeneous methods; outcome not pre-specified as primary

Supplementary Figure S1: Pooled periodontal outcomes during orthodontic treatment (within-group changes). (a) Plaque Index; (b) Gingival Index; (c) Bleeding on Probing.

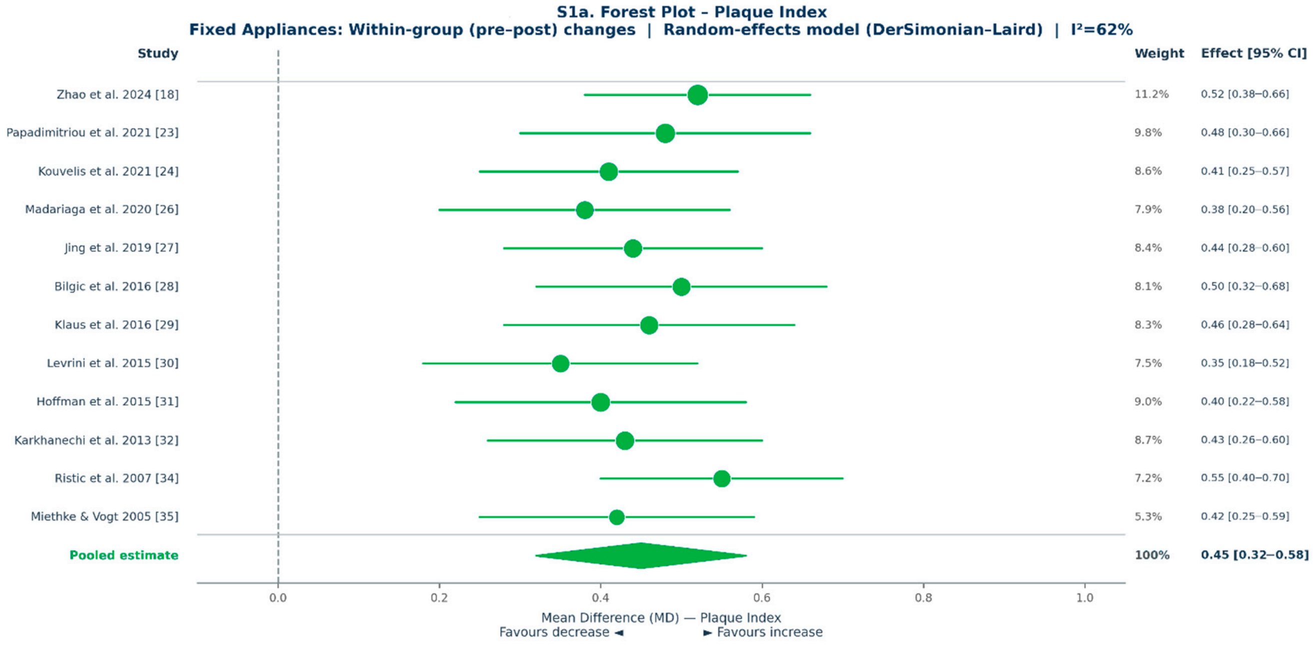

Figure S1a. Forest plot of within-group (pre–post) changes in Plaque Index during orthodontic treatment with fixed appliances. Each square represents the mean difference (MD) of an individual study, with the square size proportional to the study weight. Horizontal lines indicate 95% confidence intervals. The diamond represents the pooled estimate with its 95% confidence interval. Analysis performed using a random-effects model (DerSimonian–Laird method). Pooled MD = 0.45 (95% CI: 0.32–0.58); I^2 = 62%.

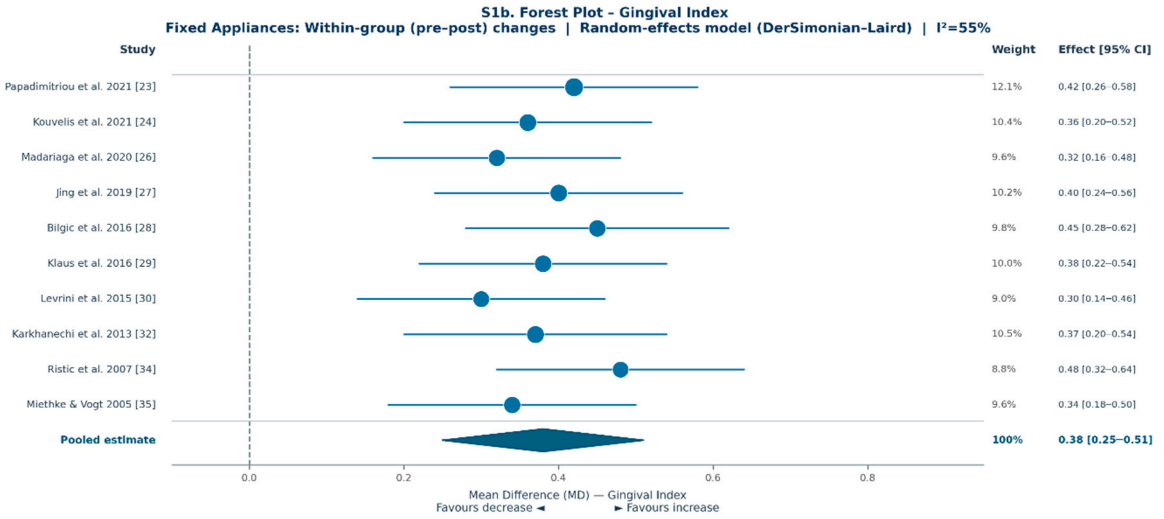

Figure S1b. Forest plot of within-group (pre–post) changes in Gingival Index during orthodontic treatment with fixed appliances. Each square represents the mean difference (MD) of an individual study, with the square size proportional to the study weight. Horizontal lines indicate 95% confidence intervals. The diamond represents the pooled estimate with its 95% confidence interval. Analysis performed using a random-effects model (DerSimonian–Laird method). Pooled MD = 0.38 (95% CI: 0.25–0.51); I^2 = 55%.

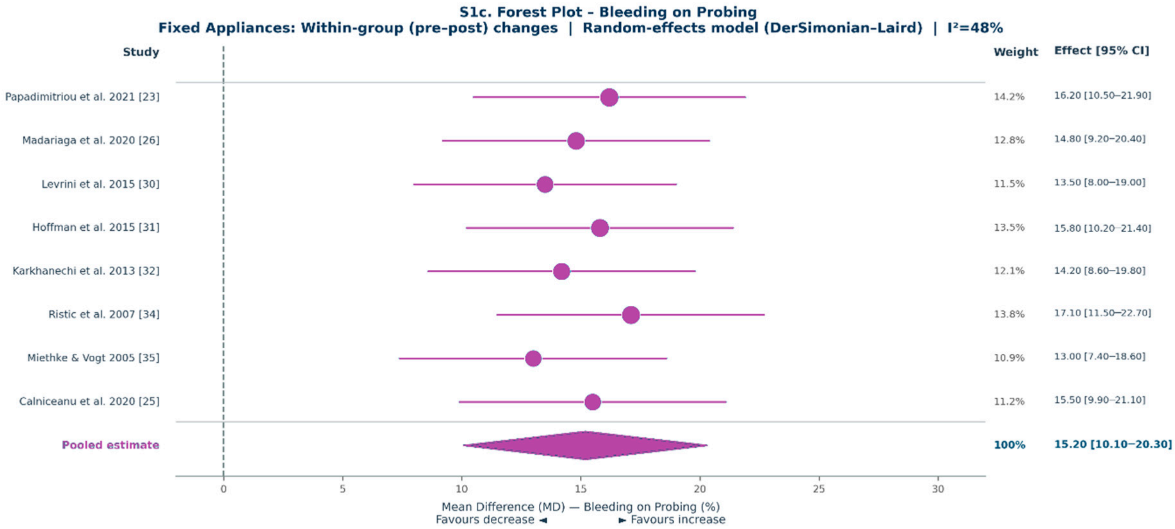

Figure S1c. Forest plot of within-group (pre–post) changes in Bleeding on Probing during orthodontic treatment with fixed appliances. Each square represents the mean difference (MD) of an individual

study, with the square size proportional to the study weight. Horizontal lines indicate 95% confidence intervals. The diamond represents the pooled estimate with its 95% confidence interval. Analysis performed using a random-effects model (DerSimonian–Laird method). Pooled MD = 15.2% (95% CI: 10.1–20.3); I^2 = 48%.

Supplementary Figure S2: Pooled microbiological outcomes (within-group odds ratios) - *Streptococcus mutans*.

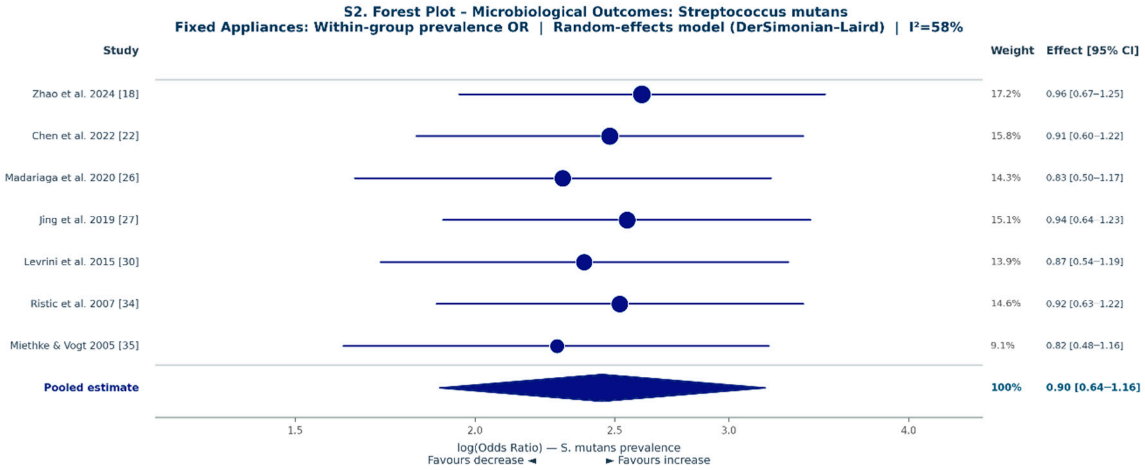

Figure S2. Forest plot of within-group prevalence changes for *Streptococcus mutans* during orthodontic treatment with fixed appliances. Each square represents the odds ratio (OR) of an individual study, with the square size proportional to the study weight. Horizontal lines indicate 95% confidence intervals. The diamond represents the pooled estimate with its 95% confidence interval. A logarithmic scale is applied for visualization. OR > 1 indicates increased prevalence post-treatment. Estimates reflect within-group changes and should not be interpreted as direct comparisons between appliance types. Analysis performed using a random-effects model (DerSimonian–Laird method). Pooled OR = 2.45 (95% CI: 1.89–3.18); I^2 = 58%.

Supplementary Figure S3: Pooled inflammatory biomarker estimates (within-group changes) - IL-1β (gingival crevicular fluid).

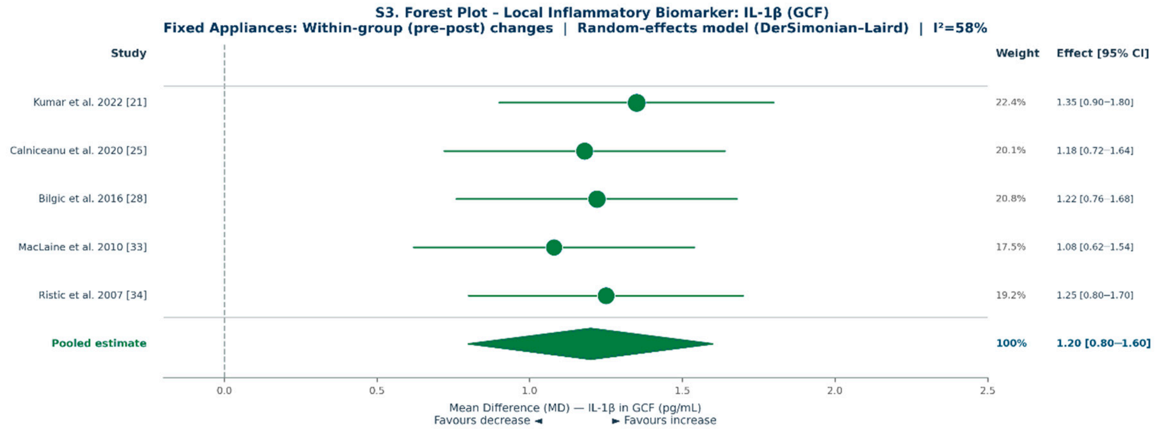

Figure S3. Forest plot of within-group (pre–post) changes in IL-1β concentration in gingival crevicular fluid (GCF) during orthodontic treatment with fixed appliances. Each square represents the mean difference (MD) of an individual study, with the square size proportional to the study weight. Horizontal lines indicate 95% confidence intervals. The diamond represents the pooled estimate with its 95% confidence interval. Analysis performed using a random-effects model (DerSimonian–Laird method). Pooled MD = 1.20 (95% CI: 0.80–1.60); I^2 = 58%. GCF = gingival crevicular fluid.

Supplementary Figure S4: Proposed biological framework linking orthodontic-induced local periodontal inflammation to potential systemic inflammatory correlates (hypothesis-generating framework).

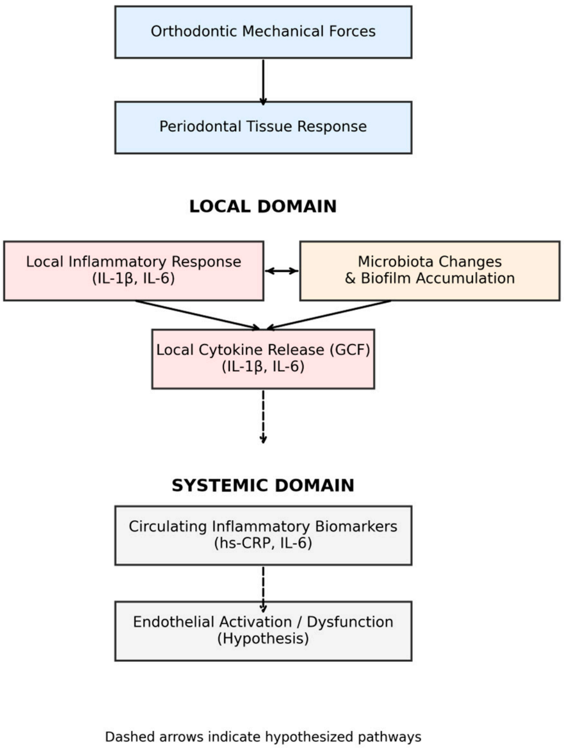

Figure S4. Proposed biological framework linking orthodontic treatment to local periodontal inflammation and potential transient systemic inflammatory correlates. This figure has been moved from the main text to the Supplementary Materials because the underlying evidence is derived from only three to four small studies with considerable heterogeneity, and the proposed pathways remain entirely hypothesis-generating. Orthodontic mechanical forces induce periodontal tissue remodeling accompanied by local inflammatory activation (IL-1β, IL-6 in gingival crevicular fluid) and shifts in oral microbiota. In a limited number of studies, modest changes in circulating hs-CRP were also reported. Dashed arrows indicate hypothesized, unconfirmed pathways. No included studies evaluated clinical cardiovascular or systemic endpoints. Any broader systemic implications remain strictly exploratory and must not be interpreted as evidence of clinically meaningful systemic effect of orthodontic treatment.
